# Multifaceted Exercise Prescription in the Management of an Overhead Athlete with Suspected Distal Biceps Tendinopathy: A Case Report

**DOI:** 10.3390/jfmk5030056

**Published:** 2020-07-29

**Authors:** Cameron Holshouser, Dhinu J. Jayaseelan

**Affiliations:** 1 Total Motion Physical Therapy, Blacksburg, VA 24060, USA; 2 Department of Health Human Function and Rehabilitation Sciences, The George Washington University, Washington, DC 20006, USA; dhinuj@gwu.edu

**Keywords:** baseball, biceps brachii, pitching, rehabilitation, tendinopathy

## Abstract

Background and Purpose: Distal biceps brachii tendinopathy is an uncommon diagnosis. Various exercise prescriptions have demonstrated efficacy in the management of tendinopathy, although studies frequently focus on the effects of a specific type of muscular contraction (i.e., concentric, isometric, or eccentric). Currently, there is limited research guiding the conservative management of distal biceps tendinopathy, particularly with overhead athletes, and even less evidence reporting a multifaceted exercise prescription for individuals with tendinopathy. The purpose of this case report is to describe the integration of various modes of therapeutic exercise into a rehabilitation program for an overhead athlete with suspected distal biceps brachii tendinopathy. Case Description: A 19-year-old male collegiate baseball pitcher presented to an outpatient physical therapy clinic via direct access for left antecubital pain, which began 6 weeks prior to the evaluation while pitching during try-outs. Following physical examination, distal biceps tendinopathy was the likely clinical diagnosis. Interventions focused on early eccentric exercise eventually progressing to concentric and plyometric activity for return to sport. Outcomes: The patient was seen five times over the course of 4 weeks. He had significant improvements of pain, patient-reported functional outcomes, global rating of change, strength, tenderness, and provocation testing. The patient was able to return to an off-season pitching program. Discussion: An impairment-based and task-specific exercise prescription was effective for this patient with distal biceps tendinopathy. Understanding the biomechanical demands of an individual’s functional limitation, in this case baseball pitching, may assist the decision-making process and optimize outcomes. Additional research into the most effective exercise prescriptions for individuals with uncommon tendinopathies is warranted.

## 1. Introduction

Distal biceps brachii tendon (DBBT) tendinopathy is a health condition infrequently seen in baseball players. DBBT injuries occur mainly in men aged 40–60 years old [1]. The typical mechanism of injury is a powerful extension force applied to the anterior aspect of the forearm with the elbow in an actively flexed position [1,2,3,4,5]. The rate of DBBT injuries have been reported to be as low as 1.2 per 100,000 people each year [1,3,6,7,8]. The biceps brachii has a double origin comprised of the long and short head, which both distally attach onto the bicipital tubercle on the proximal portion of the radius, which contribute to elbow flexion, shoulder flexion, and forearm supination motions, making the muscle essential to many functional activities [7,9].

Conservative management of DBBT tendinopathy is not well described, although exercise is the primary intervention in the rehabilitation of load-induced tendinopathy [10]. Tendinopathy typically occurs as the result of acute or chronic bouts of overload beyond tendon capacity. Identification of an individual’s baseline tendon capacity and contributing factors to dysfunction is essential to the appropriate matching of specific exercise prescriptions. Eccentric exercise has been commonly prescribed for tendinopathy, with clinical effects of decreasing pain and improving function [11]. However, despite the effective integration of exercise in many cases, some individuals with tendinopathy are reported to have persistent symptoms and reduced long-term functional outcomes [12,13]. Resultantly, it is imperative that additional investigations into best practice and treatment options are pursued for individuals with tendinopathy.

Many reports have evaluated the efficacy and/or superiority of specific types of exercise for tendinopathy (i.e., eccentric, concentric, isometric, heavy–slow resistance). In some cases, outcomes are equivocal, suggesting multiple prescription options exist for clinicians [14,15,16]. While various studies have reported pain reduction following individual contraction types [17,18], it has been suggested that short-term pain relief may not be adequate for the management of load-induced tendinopathies [19]. In the case of returning to sport-related activities, clinical decision-making should be guided by the specific tendon demands, rather than following a specific protocol. For overhead athletes, for example, strictly performing eccentric or concentric exercise may not appropriately replicate the rapid fluctuation between types of contraction. To date, the conservative rehabilitation of overhead athletes with distal biceps brachii tendon dysfunction has not been reported. Resultantly, in the absence of evidence to guide similar decision-making, application of exercise prescription principles from other tendinopathies may be useful to guide clinicians in managing their patients and clients.

The purpose of this case report is to describe the incorporation of a dynamic exercise program into a plan of care for a baseball pitcher with suspected distal biceps brachii tendinopathy.

## 2. Case Presentation

### 2.1. Case Description

A 19-year-old male collegiate baseball pitcher presented to an outpatient physical therapy clinic via direct access for left antecubital pain, which began 6 weeks prior to the evaluation. He provided consent for treatment and to publish his data, and the appropriate institutional review board deemed this case report exempt from review. While maximally throwing a curveball during tryouts, the patient heard a “pop” in his elbow. He was unable to continue pitching and took himself out of the game secondary to pain, apprehension, and lack of control. The day after the injury, he noticed bruising from the antecubital fossa into the anterior medial distal forearm, which lasted for three additional days. After the injury, he was able to throw during warm-ups, long toss 90 feet, and swing a baseball bat repetitively with minimal pain, yet he was unable to pitch off the mound. Four weeks after the injury, he tried pitching again from the mound and heard a similar painful pop while pitching. He then decided to seek physical therapy (PT) via direct access.

At initial evaluation, the patient’s primary complaint was localized pain while pitching. The patient described the pain as “sharp” initially in the antecubital fossa and anterior/medial elbow but described the pain during the evaluation as “tight” in the same locations. He pointed to his “popping” sensation at the location of the DBBT. Symptoms were generally improving with time, and pain was reported as minimal in the past 24 h. He denied any numbness, tingling, grip strength changes, instability, or hand atrophy. He denied any pain proximal to the elbow or shoulder or cervical spine pain. Aggravating factors included pitching, especially while throwing curveballs, the rapid elbow flexion component of power clean Olympic lifts and carrying/lifting heavy objects during activities of daily living. Easing factors included rest and avoiding painful activities. The patient denied seeking treatment or medical consultation prior to the evaluation. He had no remarkable past medical history or prior upper-extremity injuries. He was not taking any medications and had no history of smoking. No prior imaging was performed for this injury. The patient’s goal of PT was to screen for any serious pathology in the elbow, identify the problem, and return to pitching for the club baseball team.

### 2.2. Clinical Impression #1

Based on the subjective information, location of symptoms, and intake forms, the clinician’s primary hypothesis was DBBT tendinopathy. The patient reported a mechanism of injury consistent with substantial load on the biceps brachii (eccentric activity during rapid elbow extension and elevated stress when throwing curveballs, which require more forearm supination). The aggravating factors were also consistent with DBBT pathology due to the nature of heavy elbow flexion concentric and eccentric movement. However, the extent of DBBT injury was unclear based on subjective data.

### 2.3. Examination

A thorough physical examination was performed with the most salient findings presented in Table 1. Upon visual observation, no signs of ecchymosis, edema, hand/forearm atrophy, or Popeye deformity were noted. Seated posture included rounded shoulder and increased thoracic kyphosis. Prior to local elbow testing, proximal segments were assessed to determine the possibility of symptom referral or radiation. A neurological examination including deep tendon reflexes, myotomes, and dermatomes of the upper extremity was normal and symmetrical bilaterally. Cervical, shoulder, and scapular screening including active and passive range of motion (A/PROM) was normal and pain free. Proximal segment screening did not recreate primary elbow symptoms.

Elbow extension and flexion A/PROM was normal. Pain was provoked with passive elbow extension when combined with shoulder extension and wrist pronation (which maximally tensions the biceps brachii). Wrist A/PROM was normal in all directions. Tenderness was present approximately 2 cm proximal to the distal attachment on the radius. No palpable defect in the continuity of the DBBT was present. Increased tension was noted in the anterior/medial proximal forearm soft tissue restriction with minimal discomfort reported. No elbow joint pain, complaints of “popping” or symptoms of ulnar nerve pathology were recreated on examination.

Manual muscle testing (MMT) was performed on relevant elbow and forearm tissues. Left elbow flexion was weak and recreated typical antecubital pain when performed with the arm bent at the side and with elbow extension and shoulder flexion to 90°. Elbow extension strength was 5/5 and pain free. Left forearm supination strength was weak and recreated typical antecubital fossa pain at 90° of elbow flexion and in full elbow extension. The right elbow and forearm were strong and pain free. Wrist and hand MMT did not elicit pain or weakness.

Finally, a number of tissue differentiation tests were performed in efforts to identify the primary pathological tissue(s). Speed’s and Yergason’s tests both recreated typical DBBT pain. The biceps hook test and squeeze test were negative, making rupture less likely. Elbow valgus stress tests in multiple angles was normal. Biceps load II, full can, elbow flexion test, and Wartenberg and Tinel’s tests did not recreate typical symptoms.

### 2.4. Outcome Measures Used

The outcomes used in this study were a combination of subjective and objective measures. Pain was assessed using the numeric pain rating scale (NPRS). Focus on Therapeutic Outcomes Inc. (FOTO) was used to assess self-reported symptom impact. FOTO allows for a determination of clinically important change although psychometrics are patient specific. FOTO outcome measures and Global Rating of Change (GROC) scales have shown good validity, sensitivity, and responsiveness [20,21,22,23,24,25]. Additional outcomes were palpation, strength, and provocation testing. Informal re-evaluation was performed at each follow-up visit at the beginning of each session.

### 2.5. Clinical Impression #2

After competing diagnoses were made less likely, DBBT pathology was confirmed as the most likely cause of symptoms and functional decline. This was reinforced with a diagnostic cluster including the location of symptoms, reported mechanism of injury, positive tissue-specific provocation tests, and aggravating activities involving elbow flexion and supination. The patient did not have a DBBT rupture as the DBBT was intact (negative Hook test, biceps squeeze, reverse Popeye’s deformity). The patient did describe an audible “pop,” delayed ecchymosis, pain, and loss of function after an eccentric mechanism to the DBBT. Based on initial complaints, a partial tear was possible, however given the lack of similar signs or symptoms at evaluation, if a tear initially occurred, it was likely healing.

Based on Cook’s load-induced tendinopathy continuum [26,27], the patient initially would have fallen into the category of having a “reactive tendon” due to acute overload. At the time of the initial evaluation (6 weeks after injury), he did not demonstrate signs of inflammation and symptom irritability and severity was reduced, suggesting the tendon was less reactive than at initial onset. During the “reactive” tendinopathy phase, interventions are typically aimed at minimizing pain, which includes relatively unloading the tissue to avoid continued aggravation [26,27]. At this phase, higher tendon strain through eccentric and plyometric loading or compression through end-range stretching should be avoided. However, it was the clinician’s assessment that, at time of initial evaluation, the patient did not present with a reactive tendinopathy but rather with a tendon needing improvement in load capacity to allow for improved functional levels. Given the patient’s need for substantial eccentric control of the biceps brachii during overhead pitching and the strong evidence supporting eccentric exercise in a number of other tendinopathies, the clinician concluded that eccentric loading should be integrated as possible to optimize function.

### 2.6. Biceps Brachii Biomechanical Considerations for Baseball Pitching

When creating an optimal rehabilitation program, it is essential to understand the individual’s task-specific demands. In sports rehabilitation, mimicking sport-specific movement patterns through exercise may give the clinician a better understanding of the athlete’s readiness to play and may also give the patient more confidence in returning to sport. The overhead pitching motion consists of a sequence of movements that start in the lower extremity and trunk and transfer to the most distal segments in the upper extremity.

There are six phases of pitching: windup, early cocking/stride, late cocking, acceleration, deceleration, and follow-through. The windup is when the transfer of energy from the ground into the lower extremities and trunk occurs. The early cocking/stride phase begins once the lead leg reaches the maximum height and the ball is removed from the glove and continues as the pelvis and lead leg travel down the mound toward the home plate [28]. The late cocking phase occurs between lead foot contact and the point of maximal external rotation (ER) of the throwing shoulder. During this phase, maximum valgus force torque is experienced at the elbow [28]. The biceps brachii reaches peak activity as it flexes the elbow, limits anterior humeral translation, and provides a compression force on the humeral head [29]. Extreme amounts of glenohumeral ER are achieved at this stage. The acceleration stage starts between maximum ER and ball release [28]. Internal rotation (IR) velocities have been recorded as high as 7000° to 9000° per second [30,31]. During the acceleration, the elbow initially flexes from 90° to 120°, and then rapid extends to near 25° just before ball release [31,32]. The biceps brachii supplies elbow flexion torque reaching a maximum value of up to 61 N-m just before ball release [33]. Maximum elbow extension angular velocity occurs just before ball release and may reach as high as 2251° per second [34,35]. The deceleration phase occurs between ball release and maximum glenohumeral IR and elbow extension. This phase is typically described as the most violent phase [28]. During deceleration, there is marked biceps brachii and brachialis activity decelerating the rapidly extending elbow and pronating forearm [36]. The follow-through proceeds as the body continues the motion until motion has ended.

Many pitchers, such as the patient in this case study, have a variety of pitches that they use. Our patient primarily used fastballs, change-ups, and curveballs for his pitch selection. The curveball was the pitch that the patient stated he had increased pain with during pitching. The curveball arm motion and grip are almost identical to the fastball, but rather than gripping on the top of the ball, fingers are placed on the side of the ball during curveballs [28]. During curves, the pitcher will supinate the forearm until ball release during the late acceleration, generating rotation of the ball around a central axis. The added supination could theoretically increase biceps brachii loading, as compared to the fastball. Maximum elbow extension angular velocity and shoulder IR angular velocity are greater with a curveball versus that with a changeup [37].

### 2.7. Intervention

After taking into consideration the DBBT pathology and the sport-specific demands of overhead throwing, the primary focus of the intervention program was an impairment-based approach, with an emphasis on improving tendon load capacity while minimizing undue strain through addressing adjacent-segment inadequacies. Assessment of the patient’s function, pain, and strength was used to guide the intensity of progression toward his goal of pitching. Intervention progression is described in detail in Table 2.

Interventions initially focused on restoring pain-free AROM for the elbow and forearm using high repetitions and low load [38,39,40,41]. Soft tissue restrictions of the forearm were addressed to allow for optimal movement patterns. Instrument-assisted soft tissue mobilization (IASTM) was performed on areas of restriction and followed by self-stretching [42,43,44]. The patient was instructed to stretch the anterior forearm and biceps with elbow/wrist/flinger extension for three sets of thirty seconds each. The patient was also instructed to perform this stretch after performing a self-massage using hands of biceps and anterior/medial forearm for thirty seconds each. Education was provided to avoid aggravating activities (i.e., heavy lifting, power cleans, pitching) until the effect of treatment was established.

**Figure 1:**
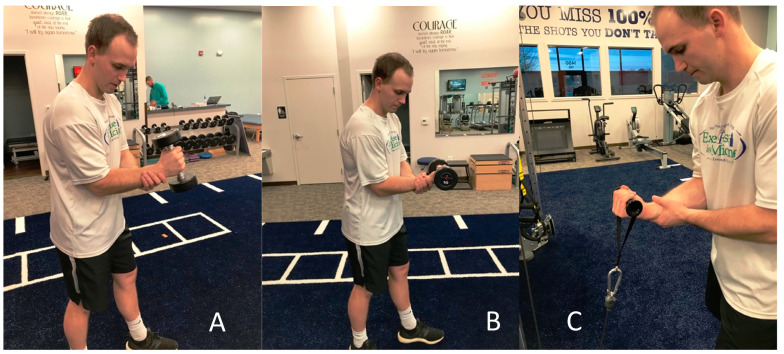
Eccentric elbow flexion with the wrist in (**A**) neutral with dumbbell, (**B**) supinated with dumbbell, and (**C**) supinated with pulley. Each image is shown using the contralateral arm as support during the eccentric lowering of the motion.

**Figure 2:**
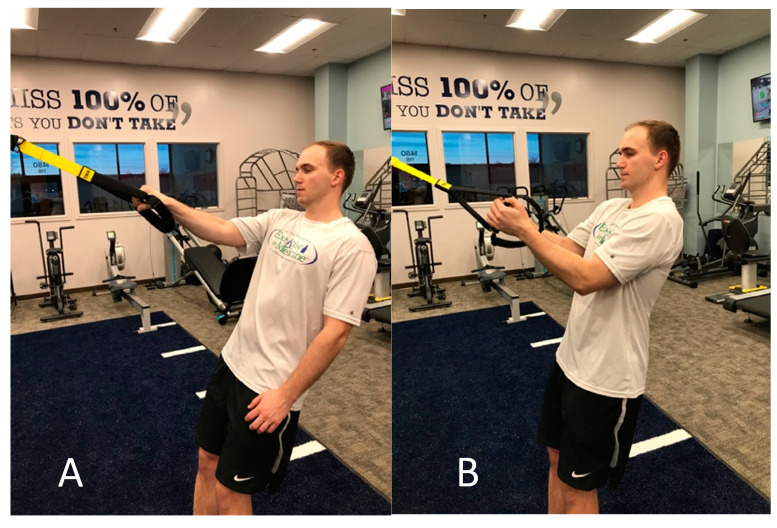
Eccentric elbow flexion with wrist in pronation using TRX ™.

Eccentric training on the first session (initial evaluation) was performed with low intensity. Multiple forearm positions were used in order to replicate the stress on the biceps during overhead pitching and to strengthen additional elbow flexors (brachialis and brachioradialis) (Figure 1 and Figure 2). In efforts to avoid undue strain and manage total tension of the tendon, eccentric training started with mid-range positions of shoulder and elbow flexion. Three sets of seven repetitions were prescribed, as this dosage was found effective for a high-level wrestler with distal biceps tendinopathy [45]. After the second session, the patient was instructed to perform the exercises every day at the gym with supinated, neutral, and pronated grip using a load that was “heavy as tolerated, feeling uncomfortable but not disabling,” with good form and able to control eccentric descent for three seconds.

Numerous variables such as rotator cuff fatigue or lack of scapular control can contribute to poor shoulder mechanics and excessive strain on the arm while pitching [46,47,48,49,50]. Shoulder ER/IR exercises were performed, starting by the side then progressing to 90° shoulder abduction and 90° elbow flexion positions. Scapular strength and endurance are essential for pitching. Scapular strengthening was performed, yet many common scapular strengthening exercises use concentric elbow flexion (i.e., rows). Exercises started in prone position to improve scapular control while avoiding pulling motions at the elbow. The patient reported that he performed eccentric training every day outside of the clinic other than two rest days.

Concentric biceps activation was emphasized two weeks after evaluation, with multiple forearm positions such as supination, pronation, and neutral grip once the patient had non-painful MMT for elbow flexion and wrist supination. The primary focus of concentric training was to improve the biceps strength in mid-range positions. Dosage for concentric training was three sets of ten repetitions, which have been shown to improve muscle strength and endurance [51]. Load was chosen based on rate of perceived exertion as the patient was instructed to select a weight that ensured the last two reps out of ten were challenging while maintaining good form. Eccentric loading was gradually increased as tolerated to build tendon capacity. Pitching specific exercises were also progressed with increased resistance and repetitions toward sport-specific positions such as arm cocking. Resistance bands were used to perform shoulder external and internal rotation due to the ability for the patient to perform easily at home as well as the end-range strengthening qualities of the band. Rows with pulley were integrated to allow for concentric elbow flexion in more functional movement patterns.

**Figure 3:**
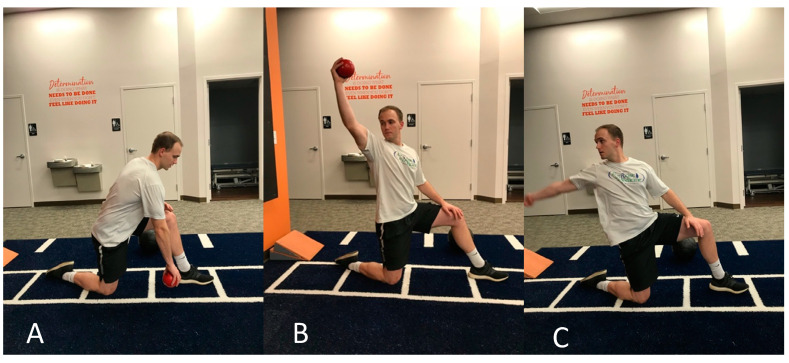
Reverse plyo-ball throw against wall working on rapid eccentric loading of biceps brachii. (**A**) Starting phase, (**B**) rapid shoulder flexion acceleration with supinated wrist, (**C**) deceleration phase after ball release.

**Figure 4:**
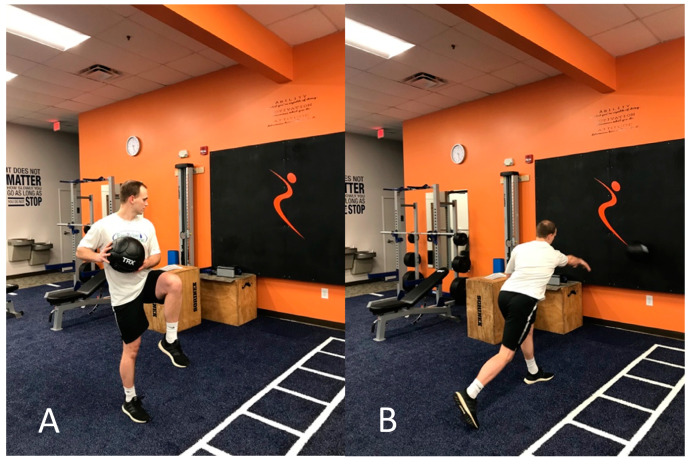
Plyometric medicine ball throw against wall. (**A**) Starting in wind-up position, (**B**) deceleration phase after ball release.

**Figure 5:**
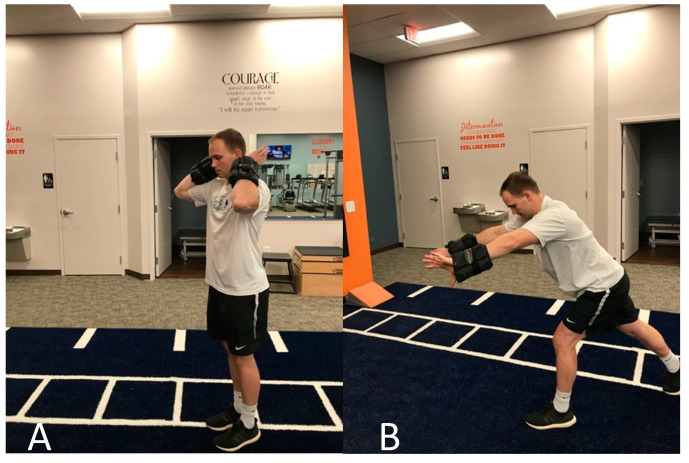
Rapid eccentric biceps brachii loading using wrist weights. (**A**) Starting position in elbow flexion and wrist supination, (**B**) end position after rapid elbow extension and wrist pronation into follow-through phase.

Once the patient had asymptomatic resisted and provocative testing, progression focused on plyometric training and baseball-specific movement patterns (Figure 3, Figure 4 and Figure 5). Exercises incorporated rapid, sport-specific eccentric loading of the DBBT. Fast concentric motions at end range were also included during this stage. After completing the plyometric exercises, the patient performed light throwing with a tennis ball at thirty feet. Unfortunately, the patient was returning home for winter break after the fifth session and was unable to continue formal PT. He was given a progressive return to throw program to be performed during the month of break before club baseball resumed. The interval long-toss throwing program started on flat ground progressing distance and number of throws. After completing a long-toss program, the program progressed to simulated pitching off flat ground and then on the mound. Curveball throwing was initiated in the later phases of the program. It was recommended that he should be able to complete a phase without compensation or pain before progressing to the next phase. The patient was instructed to contact the treating therapist should questions arise.

### 2.8. Outcomes

The patient was seen in PT five times, including the initial evaluation, over the course of four weeks. Subjective and objective reassessment measures were completed at the beginning and end of each session, with outcomes presented in Table 3. FOTO was used on the initial evaluation and discharge. The Patient’s Physical FS Primary Measure score intake improved from 83 to 98 points and demonstrated 15 points of change. Given the patient’s risk-adjustment variables and the actual intake FS score, FOTO predicted that the patient will increase in function by at least 8 points (to 91), suggesting his minimal clinically important difference (MCID) was satisfied. NPRS and GROC both improved greater than their respective MCIDs. MMT was normal and pain free, and he no longer had tenderness or pain with provocative testing. The patient was contacted via email six weeks after discharge. He reported that he did not have any residual pain or loss of function related to his elbow. He returned to pain-free throwing and was satisfied with his improvement.

## 3. Discussion

The purpose of this case report was to describe the incorporation of a dynamic exercise program into a plan of care for a baseball pitcher with suspected distal biceps brachii tendinopathy. An impairment-based program emphasizing building load capacity through eccentric, concentric, and plyometric exercise allowed improved function and reduced pain within one month and return to pitching at intermediate follow-up. Currently there is limited evidence guiding the conservative management of DBBT tendinopathy, suggesting additional research is warranted.

Tendons undergo substantial tensile strain during functional activities. For the overhead athlete, the distal biceps tendon must tolerate rapid force fluctuations and torsional stress to stabilize the arm and allow for appropriate load transference. Eccentric exercise is commonly integrated for tendinopathy, with significant improvements in pain and function often reported. While the clinical diagnosis of distal biceps tendinopathy suggested eccentric exercises may be appropriate, the throwing-specific demands on the DBBT influenced the exercise prescription. The DBBT undergoes both concentric and eccentric speeds up to 2251° per second multiple times per game [34,35]. Resultantly, once eccentric training improved the patient’s functional load tolerance and pain, the priority became improving the sport-specific demands of the involved tendon as well as the adjacent segments. It is unclear whether eccentric loading alone would have been sufficient in returning this patient to his previous level of function, although comparative trials of different loading parameters for individuals with upper-extremity tendinopathy would be interesting.

Athletes require joint mobility and stability of multiple segments simultaneously to achieve success. Tendinopathy is common among the athletic population, given the possibility of loading a tendon past its capacity without appropriate rest from frequent practice and game scenarios. Specific modes of contraction (isometric, eccentric, concentric) have been independently researched in the management of tendinopathies. Short-term pain relief may exist with isometric exercise [17,18], and various exercises have been associated with long-term pain relief and functional improvement [14,52]. However, there are no reports guiding the conservative management of an overhead athlete with DBBT tendinopathy. In this case, progressive therapeutic exercise incorporating multiple modes of contraction was effective in returning a baseball pitcher to his prior level of function.

There are limitations to this case study. As with any case report, the authors can only describe the process and outcomes and not infer causality. Imaging was not performed but may have aided in diagnosing the specific pathology. Specifically, varying degrees of tendon damage (i.e., tear versus rupture) may guide exercise progression or regression. However, clinically adapting exercise prescriptions to patient capacity and symptom irritability appears to be suggested in the absence of worsening symptoms or functional decline [26], in which case, diagnostic imaging may have more relevance. While short- and intermediate-term follow-up suggested positive outcomes, long-term follow-up was not performed. Despite the inherent limitations of the case report, it is the authors hope that this report will assist in the differential diagnosis and conservative management of overhead athletes with distal biceps brachii tendinopathy.

## 4. Conclusions

Distal biceps brachii tendinopathy is an uncommon condition, particularly in overhead athletes. There is limited evidence to guide treatment of the condition, and no reports thus far have reported the conservative rehabilitation of overhead athletes with the condition. In this case report, exercise was prescribed according to biomechanical demands of baseball pitching and matched to the patient’s capacity. Functional and symptomatic improvement was noted in at discharge and at short-term follow-up. The outcomes related to this case warrant additional investigation into the best practice for this patient population.

## Figures and Tables

**Table 1:** Examination findings.

Type of Assessment	Test(s) Performed	Result	
Observation	Postural assessment	Rounded shoulders, increased thoracic kyphosis	
	Visual inspection	No local ecchymosis, edema, atrophy, or deformity	
Neurological examination	Deep tendon reflexes, dermatome, and myotomes of UE	Normal, symmetrical	
Proximal joint screening	Cervical, shoulder, scapulothoracic A/PROM	Normal, pain free	
Range of motion	Elbow A/PROM: Flexion Extension	WNL, biceps stretch * WNL	
	Wrist/Hand A/PROM: Supination Pronation Flexion Extension	WNL	
Manual muscle testing	Shoulder:	L	R
	External rotation	4/5	5/5
	Internal rotation	5/5	5/5
	Flexion	4/5	5/5
	Elbow:	L	R
	Flexion: 90° at side	4/5 *	5/5
	0°, shoulder flexion to 90°	3/5 *	5/5
	Extension	5/5	5/5
	Wrist/Hand:	L	R
	Grip strength	WNL	WNL
	Supination: at side	4/5 *	5/5
	0°, shoulder flexion to 90°	3/5 *	5/5
	Pronation	WNL	WNL
	Flexion	WNL	WNL
	Extension	WNL	WNL
Tissue differentiation	Palpation	(+) pain at DBBT (−) defect/discontinuity	
	Speed’s and Yergason’s tests	(+)	
	Hook and biceps squeeze	(−)	
	Elbow valgus stress	(−)	
	Biceps load II	(−)	

* Denotes pain; Abbreviations: AROM—active range of motion, DBBT—distal biceps brachii tendon, PROM—passive range of motion, UE—upper extremity, WNL—within normal limits.

**Table 2:** Detailed exercise prescription.

Intervention	Visit 1 (Evaluation)	Visit 2	Visit 3	Visit 4	Visit 5 (Discharge)
Manual therapy	Pronator/flexor soft tissue restriction - Manual and IASTM anterior/medial forearm - Wrist/pronator stretch 3 × 30”	Pronator/flexor soft tissue restriction - Manual and IASTM anterior/medial forearm - Wrist/pronator stretch 3 × 30”	Pronator/flexor soft tissue restriction - Manual and IASTM anterior/medial forearm - Wrist/pronator stretch 3 × 30”	Pronator/flexor soft tissue restriction - Manual and IASTM anterior/medial forearm - Wrist/pronator stretch 1′	Not performed, no mobility restrictions noted
Exercise	Pain-Free AROM - Dowel elbow flexion/extension 60× - Supination pronation 60× @ 90 deg. elbow flexion Eccentric training - Eccentric biceps curl 3 × 10, 3# DB - TRX eccentric elbow biceps with neutral grip 2 × 10 Baseball specific - Standing ER @ 0 abduction, 3 × 20, red band	Pain-Free AROM - Rows with shoulder pulleys 2′ - Dowel elbow flexion/extension 60× - Supination pronation 60× @ 90 deg. elbow flexion - Assault bike UE and LE, light 5′ Eccentric training - 3 × 7 cable pulley eccentric on painful side 12.5#, supinated grip - 3 × 7 neutral grip curl, 10# DB - 3 × 7 TRX Eccentric biceps on left, pronated grip Baseball specific - ER/IR @ 0 Abduction 30× ea. red band - ER/IR @ 45 deg. in stride position, 30× ea. red band - ER/IR @ 90/90 in stride position, 30× ea. red band - Plank shoulder taps 3 × 10 - Prone I, W, T, I 20× ea.	Pain-Free AROM - Assault bike UE and LE, light 8′ - Dowel elbow flexion/extension 60× Eccentric training - 3 × 7 cable pulley eccentric on painful side 17.5#, supinated grip - 3 × 7 neutral grip curl, 15# DB - 3 × 7 TRX Eccentric biceps on left, pronated grip - Bent over eccentric row 20# KB Baseball specific - ER/IR @ 0 Abduction 2 × 30 ea. red band - ER/IR @ 45 deg. in stride position, 2 × 30 ea. red band - ER/IR @ 90/90 in stride position, 2 × 30 ea. red band - Plank shoulder taps 3 × 10 - Prone I, W, T, I 2 × 20 ea.	Pain-Free AROM - Assault bike UE and LE, 8′ Eccentric training - 3 × 7 cable pulley eccentric on painful side 17.5#, supinated grip - 3 × 7 neutral grip curl, 20# DB - 3 × 7 TRX Eccentric biceps on left, pronated grip Concentric training - Neutral grip DB: 3 × 10, 5# - Supinated grip cable, 3 × 10, 2.5# - Pronated grip, 3# DB 3 × 10 - Lunge follow-through position concentric cable rows 2 × 10 each leg, 7.5# Baseball specific - ER/IR @ 0 Abduction 60× ea., red, quick - ER/IR @ 90/90 2 × 20 red, quick - Push-ups 20×	Pain-Free AROM - Assault bike UE and LE, 8′ Concentric Training - Neutral grip DB: 3 × 10, 10# - Supinated grip cable, 2 × 20, 5# Baseball specific - Elbow flexion/supination to elbow extension/pronation –quick 5# wrist weight 3 × 7 - Overhead biceps flexion to elbow extension/pronation- quick 5# weights weight 3 × 7 - Reverse throw with red ball against wall (faster eccentric) 2 × 7 - Single leg kettlebell shot put throw 4# med ball 2 × 7 - Overhead med ball throw 2 × 7 - Elbow extended ball flips 2 × 15, red ball - Tennis ball throwing 2 × 20 - Pronated ball catches in flexion - Single-arm underhand toss 2 × 7
Education	- Limiting amount of heavy lifting with elbow flexion - Limiting end-range shoulder or elbow motions with quick and forceful stress on biceps tendon especially when combined with supination - No throwing	- AROM elbow flex/ext., wrist sup/pro – active pain free 2×/day 60 reps ea. - Forearm and distal biceps self-massage with hand - Wrist flexor stretch 3×/day, 2 × 30”	- AROM elbow flex/ext., wrist sup/pro – active pain free 2×/day 60 reps ea. - Forearm and distal biceps self-massage with hand - Wrist flexor stretch 3×/day, 2 × 30” - Eccentric training every day at gym with supinated, neutral, pronated grip 3 × 7 each, “heavy as tolerated, uncomfortable but not disabling, good form; pulley supinated, DB neutral, kettlebell pronated”	HEP: Eccentric training - 3×/week - Same intensity, progressing if easy add light concentric training	Return to throwing program, no curveballs for 2 weeks, should be able to perform flat ground prior to mound throwing, continue HEP, stretch and self-soft tissue massage between innings or sessions

Abbreviations: AROM—active range of motion, DB—dumbbell, ER—external rotation, HEP—home exercise program, IASTM—instrument-assisted soft tissue mobilization, IR—internal rotation, LE—lower extremity, UE—upper extremity.

**Table 3:** Subjective and objective outcomes.

Outcome Measure	Initial Evaluation	Discharge (4 weeks)
NPRS (average over past 24 h)	4/10	0/10
Patient Physical Functional Status (FOTO)	83/100	98/100
Participating in recreational activities in which you take some force or impact through your elbow, wrist, or hand (FOTO)	With Mild Difficulty	With No Difficulty
Severity of any weakness (FOTO)	Mild	None
Global Rating of Change (FOTO)	N/A	+5
Resisted biceps brachii testing	Elbow flexed: 4/5 * Elbow extended: 3/5 with pain	Elbow flexed: 5/5 Elbow extended: 5/5
Tenderness to palpation	(+) distal biceps tendon	(−)
Tissue differentiation tests	Speed’s: (+) Yergason’s (+)	Speed’s: (−) Yergason’s: (−)

* Denotes pain; Abbreviations: NPRS—numeric pain rating scale; FOTO—Focus on Therapeutic Outcomes Inc.
